# Observations on the Use of Crystallized Subacetate of Lead in Hypertrophy of the Heart

**Published:** 1856-04

**Authors:** J. L. Brachet


### RECORD OF MEDICAL SCIENCE.

### *Observations on the Use of Crystallized Subacetate of Lead in Hypertrophy of the Heart.* By J. L. Brachet.

[Paper read to the Academie de Medicine.]

When the diseases of the heart are considered, and above all aneurism, the physician can not but view with dread the terrible anathema placed at the head of the work of Corvisart, "*hæret lateri lethalis arundo.*" Experience has unfortunately but too well confirmed its truth. The efforts of Albertini, Valsalva, Læennec, Hope, Kreysig, and more recently, those of Bouillaud, have been far from producing the brilliant results promised from them. Science and humanity yet demand means of cure. Bleeding, modified in many ways, digitalis, the cyanides and other sedatives, ammonia, quinine, the preparations of iron, acetate of potassa, mineral waters, and regimen, have in their turns taken a stand and inspired hopes, which have not been fulfilled. But should this deter us from further research for the discovery of more efficacious means? Should we recoil before the many difficulties which beset our path? You at least, gentlemen, will not think thus.

I beg to submit to you a few facts concerning aneurism; I trust I may be able to call the attention of practitioners to this important subject, and to a remedial agent which has succeeded well in my hands. My experience dates back for thirty years. In 1848, in my prize essay on colica pictonum, I stated that I had made frequent use of the acetate of lead in large doses, without ever inducing any unpleasant effects. I then had reference to its use in the treatment of aneurism. Since that time I have continued to use it on all occasions that presented themselves for its exhibition, and have thus been enabled to collect many observations concerning the effect it produces. It would be an abuse of your patience to enumerate all of them at this time, more particularly as my principal desire is simply to call your attention to this plan of medication; I shall therefore confine myself to the report of a few cases.

Case 1st.—A son of M. N. had been at boarding school for several years. At the age of twelve it was first remarked that he showed symptoms of oppression in his breathing after exercise, and subsequently that the action of the heart was much increased. For nearly three years he was subjected to all rational treatment. Bleeding; the preparations of digitalis; hydrocyanic acid in all its forms; nothing seemed to answer; a few days relief would be followed by a recurrence of the symptoms. The patient was removed from school. He was thought to be affected with aneurism. He was at this time fifteen years of age. When first I saw him he presented all the signs of precocious adolescence: his heart beat with great force and over a large space; the least movement caused an increase in the palpitations and the symptoms of oppression. All his symptoms indicated hypertrophy of the left ventricle considerably advanced. I began my treatment by blood-letting to twelve ounces, enjoined repose, and ordered emollient drinks, with the cherry laurel water. A week after this his symptoms seemed to have moderated; the palpitation was less marked. The mild treatment was continued; two weeks elapsed without further improvement; digitalis was then ordered in medium doses; a month later no result from the treatment. I then thought of the sugar of lead which I had seen used by Dupuytren, and ordered it combined with digitalis in the following manner:

Plumbi Acetatis, ℈ii;
Extracti Digitalis, ℈i. m., ft. pil. No. 20.

The patient took one of these pills morning and night. Five days after beginning the treatment the action of the heart was less violent but the impulse was as diffuse as before. One pill more was ordered in the morning. At the expiration of another five days further improvement. Dose increased to two pills morning and night. This treatment was continued for two months. The patient was seen every five days. At each visit the symptoms were found to be less intense and the volume of the heart sensibly diminished.

The treatment was now suspended for fear of inducing the toxic effects of the lead. Ten days afterwards condition of patient the same. No farther treatment for some time. The patient, becoming tired of confinement, desired to take exercise. Every time that he went out he returned more and more fatigued; the effort of ascending the stairs increased the palpitation which gradually assumed a character and permanence indicative of a return of his disease; repose was again ordered and the use of the medicine recommenced. A change for the better was soon observed. The continuation of the treatment produced a diminution in the volume of the heart which was perceptible from week to week. In the course of four months the organ seemed to have returned to its normal condition, nevertheless the palpitation recurred to a certain extent upon any sudden emotion or movement. At this time, twenty-five years after treatment, the condition of the patient is good, his palpitation occurs but seldom, and during repose it is impossible to detect anything abnormal in the condition of the heart.

The favorable action of the acetate of lead seems to be well demonstrated by this case. Several plans of treatment which had been persevered in for a length of time had completely failed; the use of the lead combined with digitalis resulted in an improvement which increased steadily. When discontinued, the disease tended to resume its former gravity when recommenced; the symptoms moderated rapidly. I desire to call attention to the fact that my patient was young. This is important, for it has been during this period of life that I have had the most fortunate results from my treatment. The reason will appear hereafter.

Case 2.—Madam X., of good constitution, had enjoyed uninterrupted health up to her 25th year. At this period domestic afflictions interrupted the harmony of her life. Brooding over her sorrows made her melancholy and morose. She became nervous and experienced sensations of palpitations which seemed to augment daily. She lost her appetite and her rest was disturbed. The cardiac symptoms became more urgent and the heart's impulse began to be felt over a large space,

The functions of the lungs were interfered with and great oppression ensued. I saw her first in this condition.

The left ventricle appeared to be largely hypertrophied and the heart's action laborious. I ordered bleeding to twelve ounces and placed the patient on demulcent drinks and the cyanic preparations. This treatment persevered in for two weeks produced no effect The disease seemed to progress. The patient now went into the country where she remained a month. She returned worse. The action of her heart was much increased and its impulses extended over a larger surface. She had ceased to menstruate. Fifteen leeches were applied to the internal surfaces of the thighs; the blood flowed freely. A treatment similar to that first made use of was pursued for two weeks but with no good result. The pills of digitalis and lead were now ordered. She began by taking two a day. Every fifth day the dose of the pills was increased by one until the patient took six daily. Under the influence of this treatment the palpitation ceased by degrees, the space over which the heart's impulse was felt diminished, and at the end of four months the organ had returned to its normal dimensions.

The action of the lead in this case can scarcely be doubted. Demulcents and calmants with repose persevered in for three months produced no effect. Eight days' treatment with the lead produced a diminution in the volume and action of the heart. The improvement was uninterrupted, and at the end of four months all morbid symptoms seemed to have disappeared.

Case 3.—Madam G. has had five children two of which died very young. Endowed with a strong constitution she had always enjoyed good health until she had the misfortune to lose a son aged 19 years. While watching by his sick bed she took but little heed of herself, and the sleepless nights passed in the sick room during a rigorous season terminated in an attack of rheumatism from which she recovered with difficulty. Since that time she had experienced palpitation of the heart continually. Years passed, the palpitation increasing causing severe oppression which at times amounted almost to suffocation. During one of these attacks of difficult breathing I saw the patient for the first time. I had but little hope of effecting a cure; the organic lesion seemed to be too extensive; nevertheless the lead pills together with sedatives were prescribed and my astonishment was great when at the end of eight days I observed a great improvement, the usual condition of things re-established, and my patient resuming her ordinary avocations. Ten months afterwards and without any apparent cause she suffered another attack of suffocation. This was accompanied with slight œdema of the feet. I had immediate recourse to the treatment before pursued and in less than two months she was comparatively well. Since that time the patient has twice had an exacerbation of her symptoms and on each occasion the treatment has been successful in establishing the usual order of things. The last attack was so severe that I was doubtful of her recovery; there was] considerable effusion into the serous cavities, which, with other symptoms, gave rise to the most serious apprehension. Diuresis was established and absorption of the effused liquid was affected, the oppression however remained; the pills of lead and digitalis were ordered and their use continued for three weeks, when her condition was so much improved that she was able to walk to my house to thank me for the relief afforded her.

I shall confine myself to the relation of these few cases. They appear to me sufficient to inspire confidence in the efficacy of the subacetate of lead in the treatment of the affection in question. Were it necessary I could add many other cases, but it is scarcely requisite, they would only confirm the results which I have detailed. It must not however be supposed that my success had invariably been good. Let me hasten to disabuse you of this opinion. I have frequently been unsuccessful. I have on such occasions succeeded only in producing a temporary amelioration. These cases have usually been of hypertrophy of long standing, and it would only be to destroy confidence in the remedial means recommended, to make use of them in such cases with the expectation of a permanent cure. But in cases of recent hypertrophy my success has invariably been good.

It may be asked in what manner the acetate of lead acts in these cases. On this point I am forced to declare my ignorance. All that can be possibly said, judging from its direct action on the tissues, is that it determines a species of astringency which favors the contraction of the capillaries of the organ and produces an absorption of the hypertrophied molecules. When the acetate of lead, properly diluted, is applied to the skin or any other tissue, a remarkable styptic effect is seen, which is specially observable upon the buccal mucous membrane. The tissues contract and becomes rugose; it is rendered pale; and in case inflammation exists resolution follows. That which takes place in parts exposed to the eye may occur in deep seated organs. But here an objection arises. The acetate of lead has never been observed to produce any effect upon other deep seated organs of the economy affected with hypertrophy, and from this we would be led to infer that its astringent and resolutive effects would be wanting in hypertrophy of the heart. This reasoning would be just and sufficient did we not know that certain agents have a specific action on certain organs. I believe firmly in the special elective action of the lead upon the heart as much as I do that mercury acts upon the salivary glands, cantharides on the bladder, or diuretics on the kidneys.

It will be understood why the operation of the medicine is more prompt and effectual in young subjects. In them the movement of the integral molecules is effected more readily; the nutritive action is much greater.

If, however, this interpretation of the effect of the remedy should meet with opposition, let me declare that I attach but little importance to it.

I thank you for the honor which you have accorded me in permitting me to communicate directly to you a few of the cases in which I have employed the sugar of lead. My object, as I stated before, has been simply to call the attention of yourselves as masters of the art of the most important point in the theapeutic treatment of one of the gravest of diseases.—*(New Orleans) Medical News and Hospital Gazette.*

NOTE.—Acetate of lead was employed by Professor Wood, in hypertrophy of the heart as far back as the year 1842, to our own knowledge. When in the Pennsylvania Hospital, we not only frequently heard him inculcate its use as a most efficient means for lessening the size of the heart, but saw it prescribed in several instances. On reference to his Practice, we find the following remarks concerning it: "I have employed acetate of lead with a view to its sedative as well as astringent action, and have seen the heart apparently diminished in size under its influence; but in order to do good, it must be continued long, being suspended when it produces any symptoms of gastric or intestinal disorder, and resumed when these have ceased."—EDITOR.

